# Microscopic transorbital vs mini-pterional approach to MCA bifurcation aneurysms: a quantitative cadaveric comparative study with surgical implications

**DOI:** 10.1007/s00701-026-06821-7

**Published:** 2026-03-06

**Authors:** Beste Gülsuna, Xiaochun Zhao, Stefen Dollar, Burak Özaydin, Andrew M. Bauer, Ian F. Dunn, Walter C. Jean, Christopher S. Graffeo

**Affiliations:** 1 Department of Neurosurgery, University of Oklahoma College of Medicine, Oklahoma City, OK USA; 2 Division of Neurosurgery, Lehigh Valley Fleming Neuroscience Institute, Allentown, PA USA; 3 Department of Neurosurgery, University of Oklahoma Health Sciences Center, OKC, 1000 N Lincoln Blvd, #4000, Oklahoma City, OKC 73104 USA

**Keywords:** Aneurysm, Middle cerebral artery, Transorbital approach, Transpalpebral approach, Cadaveric analysis

## Abstract

**Objective:**

The mini-pterional (MP) approach is widely adopted as the standard exposure for middle cerebral artery (MCA) aneurysms, whereas the eyelid transorbital (TOA) approach has recently emerged as a minimally invasive alternative. This cadaveric study aims to quantitatively compare the anatomical exposure and working geometry of the mini-pterional and microscopic transorbital approaches to the MCA bifurcation, with implications for approach selection and skull base microsurgical planning.

**Methods:**

Five latex-injected human cadaveric heads were dissected via either the MP (*n* = 5) or TOA (*n* = 5) approach. Standardized microsurgical techniques of eyelid transorbital and mini-pterional approaches were used to access the MCA bifurcation. Key surgical parameters including access depth, access angle (M1 angle) to first segment of MCA (M1), and horizontal and vertical angles of attack to the MCA bifurcation were evaluated quantitatively using neuronavigation. Statistical analysis was performed using the Wilcoxon Rank Sum test with a significance threshold of *p* < 0.05.

**Results:**

Both approaches provided adequate exposure to the MCA bifurcation and its branches. The working distance is similar in both approaches (MP 26.9 ± 7.48 mm vs. TOA 31.0 ± 7.49 mm, *p* = 0.24). The MP approach offered significantly wider vertical angle of attack (86.1 ± 34.82° vs. 45.3 ± 32.11°, *p* = 0.02) while the horizontal angle (25.0 ± 8.56° vs. 33.6 ± 15.33°, *p* = 0.42) was comparable in both approaches. The TOA approach demonstrates a more perpendicular M1 angle (54.3 ± 17.37° vs. 32.7 ± 17.2°, *p* = 0.03), which may be anatomically favorable for proximal control.

**Conclusion:**

The MP approach remains advantageous for broader exposure and maneuverability; however, the microscopic TOA offers distinct anatomical exposure characteristics. These findings define the geometric differences between the two routes and may inform surgical planning in selected MCA bifurcation aneurysm cases.

## Introduction

The transorbital approaches (TOAs) are a group of minimally invasive keyhole techniques that access the anterior cranial fossa via an incision hidden within the upper eyelid crease, offering excellent cosmetic outcomes and minimal tissue disruption [3, 5]. While TOAs vary in execution, they typically involve removal of the lateral orbital rim and drilling of the posterior orbital wall lateral to the superior orbital fissure, thereby creating a corridor to the anterior and middle cranial fossae [4, 8, 9]. Among these variants, a one piece modified orbitozygomatic craniotomy can further enhance the working corridor and degrees of freedom by extending the craniotomy and drilling the greater sphenoid wing [7, 14, 40, 47].

Although TOAs have shown promising results for select skull base lesions, their application to vascular pathologies remains limited and technically demanding [4, 5, 8]. While TOAs have been widely adopted in endoscopic skull base surgery, their use as a purely microscopic, microsurgical approach has not been well characterized. The corridor is constrained medially by the globe, restricting maneuverability of instruments within the operative field [18–20]. TOAs have been used in anterior circulation aneurysms, such as those involving the anterior cerebral artery and ophthalmic segment, but reports on MCA aneurysms are sparse [6, 18–20, 47].

MCA bifurcation aneurysms account for 20% of all intracranial aneurysms, and management require early M1 segment control and safe dissection around M2 branches and perforators [29, 36]. While the pterional approach remains the gold standard, the MP variant provides a less invasive alternative with preserved exposure. Given its anterolateral trajectory and focused access, the microscopic TOA has been proposed as a potential alternative route for MCA bifurcation aneurysm clipping [3, 18, 20]. However, the feasibility and limitations of this approach remain poorly defined, particularly regarding the operative view, surgical freedom, and access of proximal control when compared to the established MP approach.

Despite MP being the established gold standard, evaluating the technical feasibility, operative angles, and surgical freedom of TOA is important to determine whether it can safely offer advantages in select scenarios without compromising proximal control. While other keyhole variants, such as supraorbital and orbitozygomatic approaches, have been compared to the pterional approach for MCA aneurysms, quantitative data specifically evaluating the microscopic eyelid TOA is lacking, particularly for metrics relevant to M1 proximal control [1, 2, 39]. In this cadaveric study, we aim to address this gap by evaluating both approaches in terms of working distance, angles of access, and maneuverability for proximal control, providing surgeons with evidence-based guidance for selecting the most appropriate approach in carefully chosen patients.

## Methods

Anatomical dissections were performed on five lightly embalmed human cadaveric heads at the University of Oklahoma Surgical Innovations Laboratory. The internal carotid arteries (ICAs) and jugular veins were cannulated and injected with red and blue silicone (Dow Corning, Midland, MI, USA) to enhance vascular visualization. Lightly embalmed cadavers were chosen to better simulate tissue pliability and allow more realistic retraction, particularly of the globe, compared with fully embalmed specimens.

Two surgical approaches were evaluated: the MP approach and a variant of the TOA. In this study, the TOA is defined as a microscopic eyelid transorbital approach with a modified one-piece orbitozygomatic craniotomy. This technique corresponds to the “lateral TOA” described in the literature, which entails partial orbital rim disconnection and is thus distinct from conventional endoscopic-only transorbital approaches [7, 14, 40]. The approach has previously been characterized in detail by our group for the management of anterior circulation aneurysms, providing a technical foundation for its application in the present study [47, 48]. Five dissections were performed for each approach. Bilateral dissections were conducted in each cadaver, with the MP approach performed on one hemisphere and the TOA on the contralateral hemisphere, alternating sides across specimens to minimize potential bias.

All dissections were performed under an operating microscope (Provido, Carl Zeiss Meditec AG, Jena, Germany) following standard microsurgical principles. For each approach, the internal carotid artery (ICA), M1 segment, and M2 branches of the middle cerebral artery (MCA) were systematically identified and exposed, with particular attention to the proximal M1 segment and bifurcating M2 trunks. Stepwise documentation of each dissection was achieved using a Canon EOS R6 digital camera (Canon Inc., Tokyo, Japan) for superficial anatomy and the microscope’s integrated imaging system for deeper structures. To ensure transparency, post-acquisition image processing was limited solely to adjustments in brightness and contrast; no structural modifications were performed.

### Quantitative measurements

The TOA and MP approaches were compared in terms of working distance, surgical maneuverability (horizontal and vertical angles of attack), proximal control (access angle to M1), and area of freedom. Key anatomical landmarks were recorded as three-dimensional spatial coordinates (x, y, z in mm) using a neuronavigation system (StealthStation S8, Medtronic, Minneapolis, MN, USA). Each measurement was repeated three times per approach, and the mean value was used for analysis to reduce variability.

Statistical analysis was performed using PASW Statistics 18.0 (IBM, Armonk, NY, USA). Comparisons between approaches were conducted using the Wilcoxon Rank Sum test. Outliers were evaluated via z-scores; no data points were excluded, as all values were within ± 3. Confidence intervals and effect sizes (Cohen’s d) were calculated, with *p* < 0.05 considered statistically significant. Graphs were generated using Prism (GraphPad Software, San Diego, CA, USA).

### Working distance

To quantify the working distance, three points defining the edges of the craniotomy (rostral, caudomedial, and caudolateral) were used to create a reference plane representing the craniotomy surface. The center of the MCA bifurcation was identified as a single point in three-dimensional space. The perpendicular distance from the MCA bifurcation to this plane was calculated using the Pythagorean theorem formula by the following steps:

1. Using 3 points on the craniotomy, the equation of the plane was established:
  $$\mathrm{Plane}: a\times x+b\times y+c\times z+d=0$$ 
2. The coordinates of the MCA bifurcation were acquired: $\left({x}_{0},{y}_{0},{z}_{0}\right)$;
3. The distance was calculated with the formula:
  $$\text{Distance }= \left|\frac{a\times {x}_{0}+b\times {y}_{0}+c\times {z}_{0}+d}{\sqrt{{a}^{2}+{b}^{2}{+c}^{2}}}\right|(\mathrm{mm})$$ 

### Surgical maneuverability

Surgical freedom was assessed by measuring the angle of attack, defined as the maximal angular range through which an instrument could be manipulated at the target site in both the horizontal (mediolateral/axial) and vertical (rostrocaudal/sagittal) planes. To simulate typical instrument handling, the distal end of a 140-mm straight dissector was placed at the MCA bifurcation, and the instrument was pivoted to its maximal extent in each plane without displacing surrounding tissue. The resulting angles were calculated using the Law of Cosines, providing an objective measure of the maneuverability afforded by each approach.

$$\theta ={\mathrm{cos}}^{-1}\left(\frac{{a}^{2}+{b}^{2}-{c}^{2}}{2ab}\right)$$

The area of freedom (deg^2^) was then calculated by multiplying these two angles, providing an objective estimate of the angular workspace available for instrument handling. This method allowed direct, quantitative comparison of maneuverability between the TOA and MP approaches.

### Proximal control evaluation

Proximal control was assessed by measuring the M1 angle at a point 1 cm proximal to the MCA bifurcation. The craniotomy center was used as the base point, and a 140-mm straight dissector was positioned with its distal tip at the M1 target. The angle formed between the vector from the craniotomy center to the M1 target was then calculated relative to the horizontal and vertical planes. Spatial coordinates of the craniotomy center and M1 target were recorded using the neuronavigation system, and the angle between vectors was determined using the Law of Cosines:

$$\theta ={\mathrm{cos}}^{-1}\left(\frac{{a}^{2}+{b}^{2}-{c}^{2}}{2ab}\right)$$

where $a$ and $b$ represent the lengths of vectors from the base point, and $c$ is the distance between their endpoints. This approach provided an objective, quantitative comparison of the angles of approach to M1 between the TOA and MP approaches.

## Results

### Standardized mini-pterional technique

**Fig. 1:**
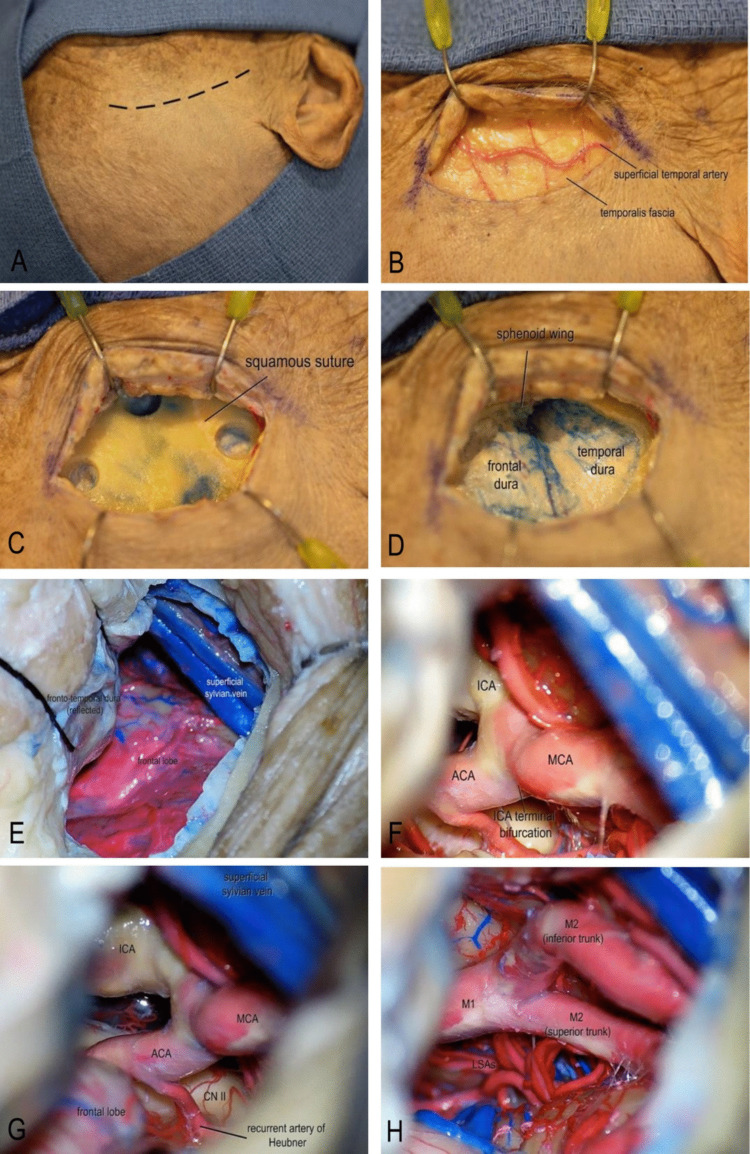
Step-by-step illustration of the standardized mini-pterional approach. **A **A curvilinear frontotemporal skin incision centered over the pterion was made. **B** After skin incision, the superficial temporal artery and temporalis fascia were exposed, and the temporalis muscle was incised parallel to the skin incision to expose the underlying bone. **C** Three burr holes were placed. **D** A mini-pterional craniotomy measuring approximately 2.5–3 cm in diameter was fashioned using a high-speed drill, and the greater sphenoid wing was drilled down to the level of the meningo-orbital band to optimize the surgical corridor. **E** The dura mater was opened in a C-shaped fashion and reflected anteriorly. **F** Standard microsurgical techniques under high magnification were used to split the Sylvian fissure and access the optico-carotid cistern. **G** Dissection of the optico-carotid cistern allowed visualization of the optic nerve, supraclinoid ICA, ICA bifurcation, ACA, and the ipsilateral recurrent artery of Heubner. **H** Distal dissection of the ICA enabled identification of the M1 segment of the MCA, MCA bifurcation, and proximal M2 opercular branches. (*ACA, anterior cerebral artery; CN II, optic nerve; ICA, internal carotid artery; M1, first segment of the middle cerebral artery; MCA, middle cerebral artery; LSAs, lenticulostriate arteries*)

Each specimen was positioned in a Mayfield skull clamp with approximately 30° of contralateral head rotation and mild extension to align the trajectory perpendicular to the ipsilateral Sylvian fissure and facilitate a low anterior corridor. A curvilinear frontotemporal skin incision centered over the pterion was placed measuring approximately 7 cm within the hair line. A single layer myocutaneous flap was employed (Fig. 1A-C).

A mini-pterional craniotomy, measuring approximately 2.5–3 cm in diameter, was fashioned using a high-speed drill and completed with rongeurs. The greater sphenoid wing was aggressively removed to the level of the meningo-orbital band to optimize the surgical corridor (Fig. 1D). The dura mater was opened in a C-shaped fashion reflected anteriorly. Standard microsurgical dissection technique was conducted under high magnification microscope to access the Sylvian fissure and optico-carotid cistern (Fig. 1E-F). The fissure was split using alternating sharp and blunt techniques, ensuring preservation of superficial Sylvian veins. Key neurovascular landmarks, including the M1 segment of the MCA, the MCA bifurcation, and the proximal M2 opercular branches. Dissection of the optico-carotid cistern permitted visualization of the optic nerve, supraclinoid ICA, and ICA bifurcation (Fig. 1G-H).

The working distance was 26.9 ± 7.48 mm. The mean vertical angle of attack was measured at 86.1 ± 34.82°, the horizontal angle of attack averaged 25.0 ± 8.56°. The M1 angle was 32.7 ± 17.19°.

### Eyelid transorbital technique

**Fig. 2:**
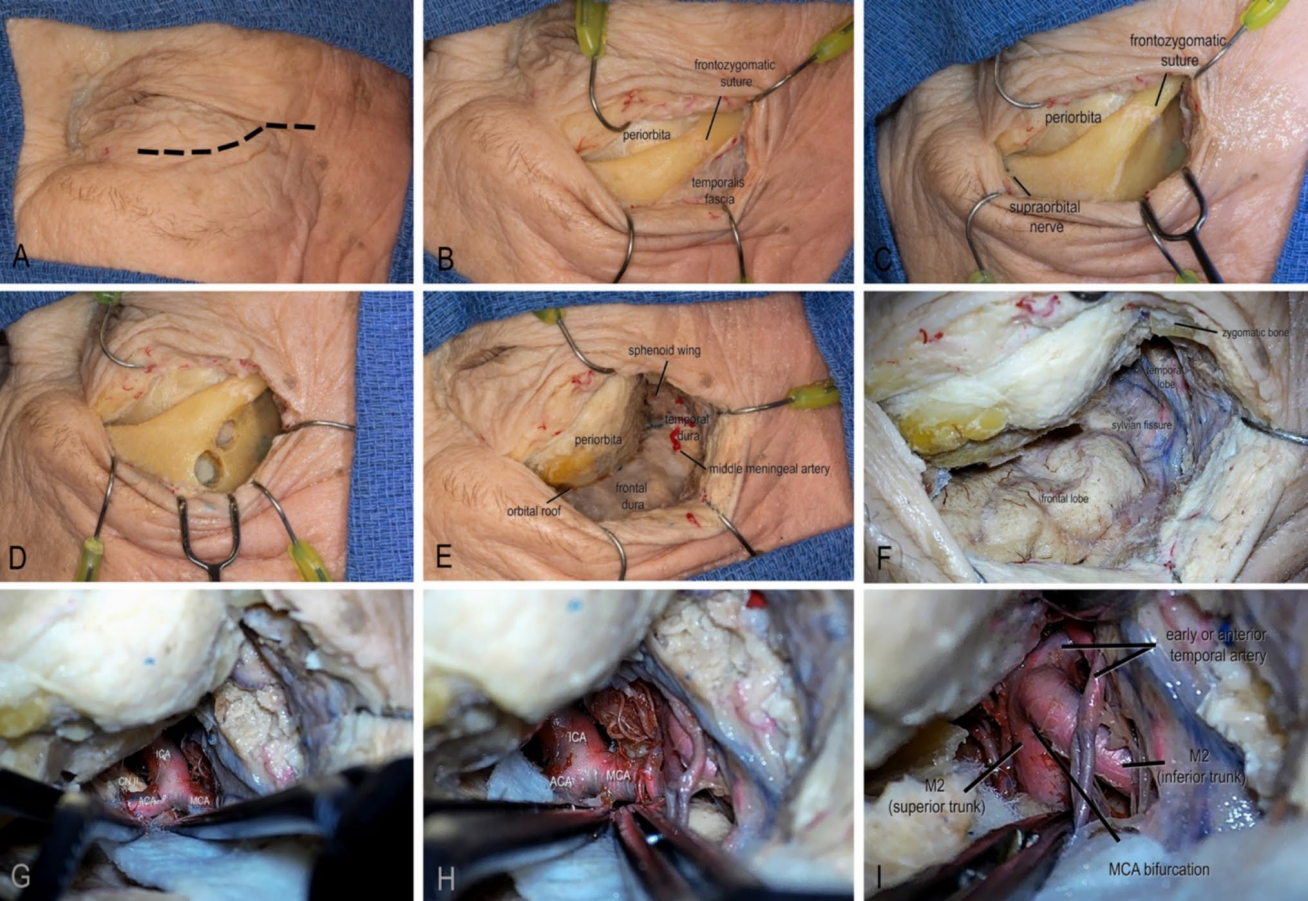
Step-by-step illustration of the eyelid transorbital approach. **A** The incision was planned along the natural eyelid crease, extending laterally from the medial limbus to the lateral canthus, up to 1.5 cm. **B** A subperiosteal dissection was carried out over the orbital rim, superior to the orbital septum. **C **The temporalis muscle was released and mobilized laterally, while the periorbita was retracted medially and inferiorly with malleable retractors to obtain adequate bony exposure for a modified one-piece craniotomy. **D** MacCarty keyholes were placed between the periorbita and frontal dura, with the orbital roof separating the two. A one-piece craniotomy was completed, with the supraorbital nerve marking the medial boundary. **E** To further expand the surgical corridor, the greater and lesser sphenoid wings were drilled. The meningo-orbital band marked the depth of the lesser wing osteotomy. The greater wing was removed until sufficient temporal dural exposure was achieved. **F** After dural opening, the Sylvian fissure, frontal lobe, and temporal lobe were directly visualized. **(G)** Using standard microsurgical techniques, the Sylvian fissure was split, providing access to the optico-carotid cistern and visualization of the supraclinoid ICA, ipsilateral optic nerve, ICA bifurcation, ACA, and MCA. **H** Further dissection exposed the ICA bifurcation, M1 segment, and MCA bifurcation. **I** The MCA bifurcation, M2 trunks, and the anterior temporal artery were visualized. (*ACA, anterior cerebral artery; CN II, optic nerve; ICA, internal carotid artery; M1, first segment of the middle cerebral artery; MCA, middle cerebral artery*)

The transorbital approach was performed via a curvilinear incision within the natural supratarsal skin crease, extending laterally from the medial limbus to the lateral canthus (Fig. 2A). A lateral extension of the incision was made within 1.5 cm distance to ensure the safety of the temporal branch of the facial nerve [3]. Subcutaneous dissection followed the orbicularis oculi fibers to expose the lateral orbital rim. Subperiosteal dissection was carried out on the orbital rim superior to orbital septum, maintaining the integrity of which can avoid violation of the orbital fat and to protect critical periorbital structures including the levator palpebrae superioris, superior rectus, and associated neurovascular bundles (Fig. 2B).

The temporalis was released and mobilized laterally and the periorbita was mobilized medially and inferiorly with malleable retractors; by doing so, adequate bony exposure was obtained to perform a modified one-piece orbito-zygomatic craniotomy (Fig. 2C). A one-piece modified orbito-zygomatic craniotomy was planned to achieve maximal exposure, A MacCarty keyhole was placed to access both frontal dura and periorbita (Fig. 2D). The craniotomy cut was made, and the lateral orbital rim was disconnected by a cutting burr with foot plate. The orbital roof was lastly fractured with a chisel to complete the craniotomy.

To further expand the working corridor, the greater and lesser sphenoid wing was extensively drilled. The meningo-orbital band marked the depth of the lesser sphenoid wing osteotomy. The greater wing was removed until adequate temporal dura was exposed (Fig. 2E). An anterior clinoidectomy can be completed in this approach, however, was not necessary in managing MCA aneurysms. Upon dural opening, the frontal lobe was gently elevated using gravity-assisted retraction (Fig. 2F). With standard microsurgical technique, the optico-carotid cistern was accessed, the supraclinoid ICA and optic nerve were visualized; The ICA bifurcation, M1, and subsequently, MCA bifurcation were exposed (Fig. 2G-I).

In the TOA approach, the working distance was 31.0 ± 7.49 mm. The horizontal and vertical angles of attack were demonstrated to be 33.6 ± 15.33° and 45.3 ± 32.11°, respectively. The M1 angle was 54.3 ± 17.37°.

### Comparative qualitative and quantitative analysis

**Fig. 3:**
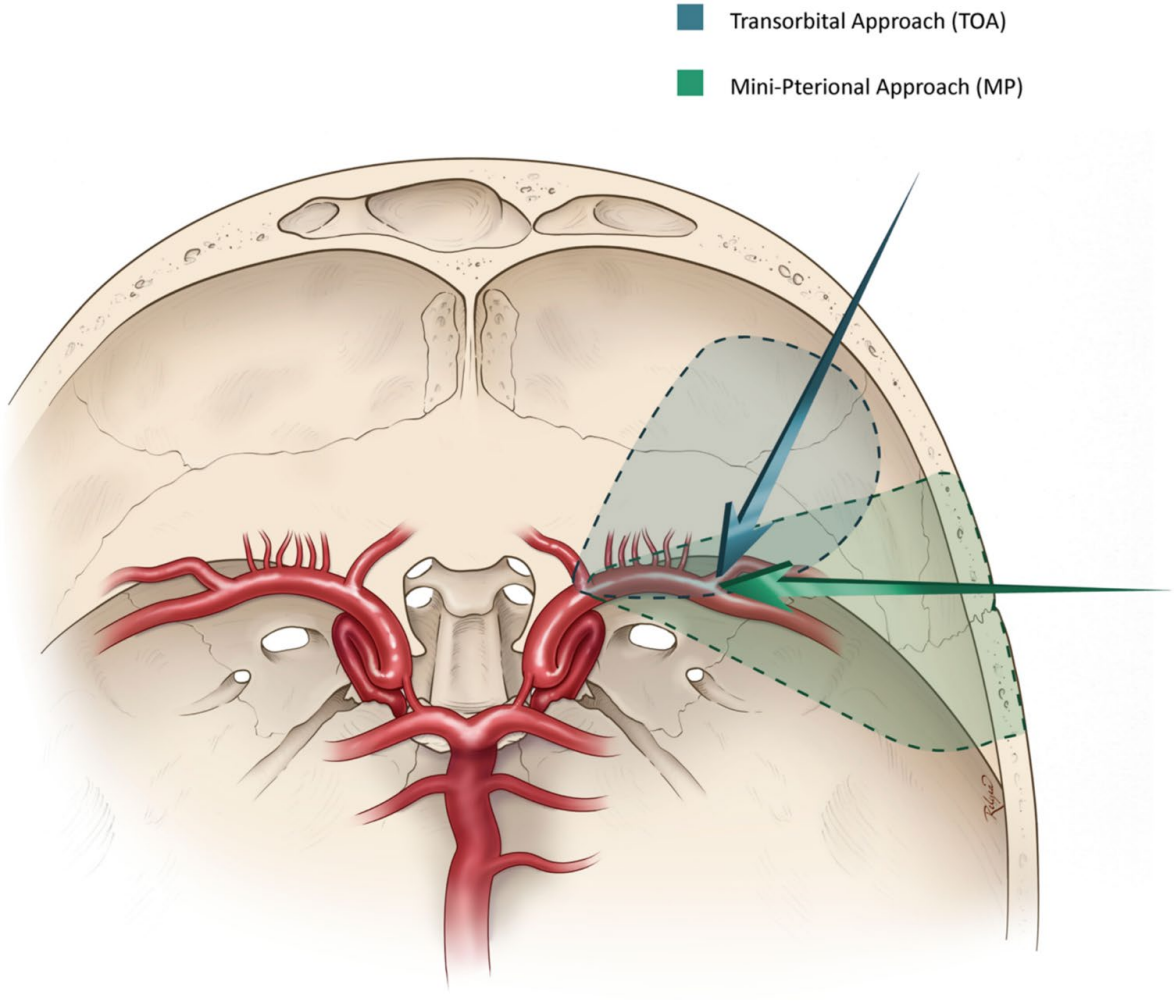
Axial illustration demonstrating the MP and TOA approaches and their respective angles to the MCA bifurcation

Subjectively, although narrower, the TOA approach provides a different unique perspective with exposure to all critical landmarks in accessing the MCA bifurcation aneurysms. With an imaginary MCA aneurysm, the access view to the M1 (proximal control) is more perpendicular as the approach is more antero-posteriorly oriented (Fig. 3).

**Fig. 4:**
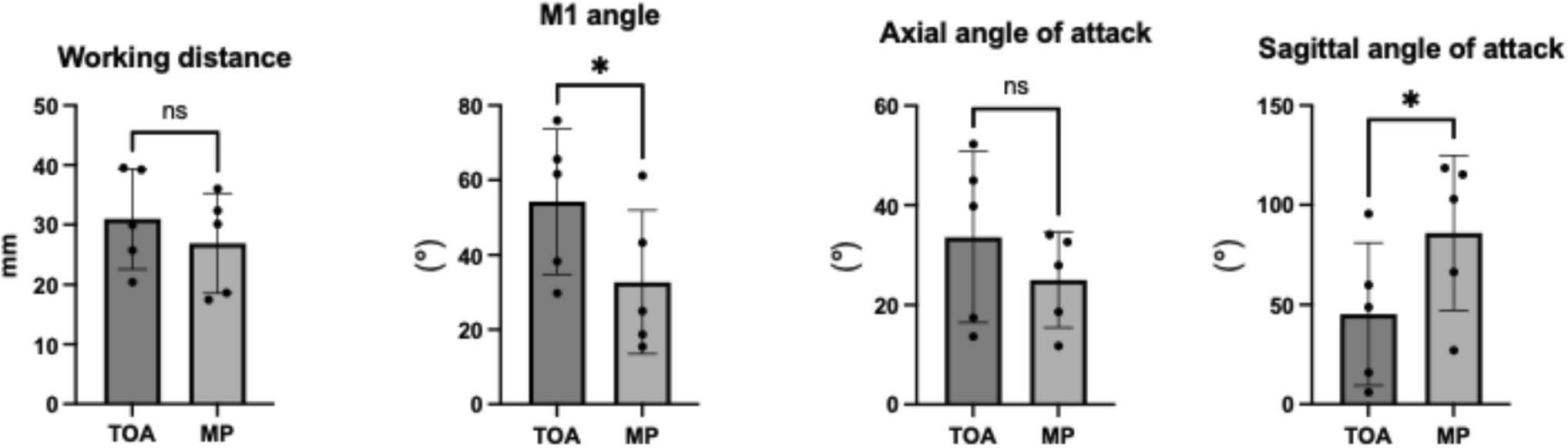
Quantitative analysis represented as bar charts. * Indicates statistically significant difference, *p* < 0.005. (*TOA* = *transorbital approach, MP* = *mini-pterional approach*)

Quantitatively (Table 1), the MP approach demonstrated greater surgical freedom, reflected by wider mean vertical angle of attack (86.1 ± 34.82° vs. 45.3 ± 32.11°, *p* = 0.02). The horizontal angle of attack (25.0 ± 8.56° vs. 33.6 ± 15.33°, *p* = 0.42) and working distance (26.9 ± 7.48 mm vs. 31.0 ± 7.49 mm, *p* = 0.24) were comparable. The TOA approach demonstrated a larger M1 angle (54.3 ± 17.37° vs. 32.7 ± 17.2°, *p* = 0.03), indicating a more perpendicular geometric access trajectory to the M1 trunk (Fig. 4).

**Table 1:** Comparison of surgical metrics

Metric	Mini-Pterional (mean ± SD)	Microscopic Eyelid Transorbital (mean ± SD)	Mean Difference (95% CI)	*p* value	Effect Size (Cohen’s d)
Sagittal/Rostrocaudal Angle (°)	86.06 ± 34.82	45.28 ± 32.11	+ 40.38 [17.71, 63.05]	**0.02**	2.21
Axial/Mediolateral Angle (°)	25.03 ± 8.56	33.61 ± 15.33	+ 8.58 [− 13.63, + 30.80]	0.42	0.59
M1 Exposure Angle (°)	32.71 ± 17.19	54.25 ± 17.37	+ 21.53 [2.02, 41.04]	**0.03**	1.25
Working Distance (mm)	26.92 ± 7.48	30.98 ± 7.49	+ 4.05 [− 10.90, + 2.81]	0.24	0.57
Area of Freedom (sq. degrees)	2152.6 ± 1141.7	1521.9 ± 1283.8	+ 630.7 [− 4970.2, + 6231.6]	0.80	0.52

### Illustrative case

**Fig. 5:**
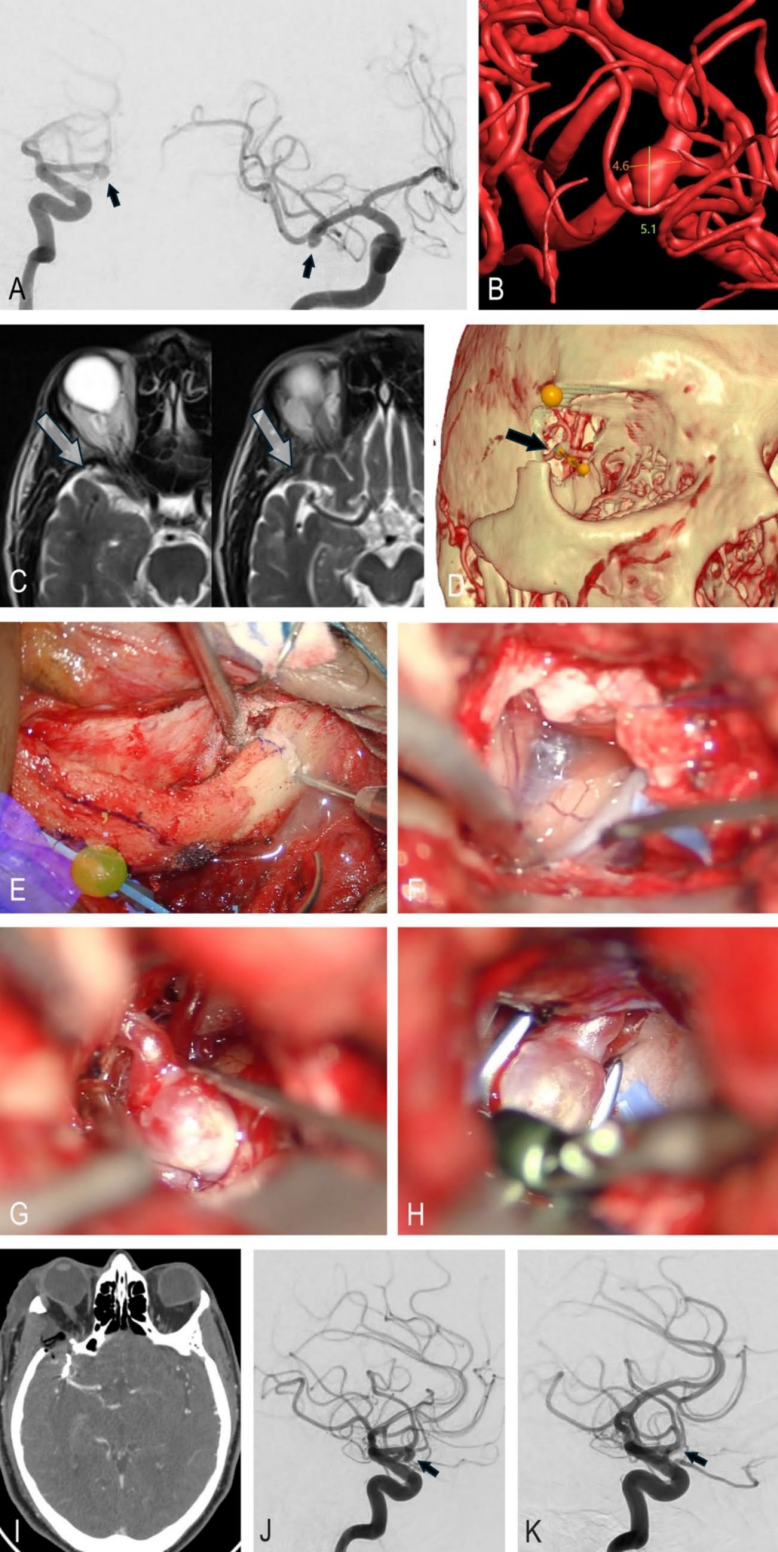
Illustrative case. **A** Preoperative lateral and anteroposterior DSA showing a right MCA bifurcation aneurysm (black arrows). **B** 3D reconstructed aneurysm model with size measurements and anticipated orientation via the transorbital approach. **C** Preoperative T2-weighted axial MRI demonstrating a wide Sylvian fissure adjacent to the sphenoid wing (arrow). **D** 3D skull and vascular reconstruction illustrating the planned transorbital trajectory (black arrow indicates aneurysm). **E** Eyelid incision and exposure of the orbital rim. **F:** Removal of greater and lesser sphenoid wings with dural opening and Sylvian fissure dissection exposing M1. **G** Aneurysm exposure with temporary M1 clip placement. **H** Definitive aneurysm clip placement. **I** Postoperative axial CT showing craniotomy site and clip position. **J–K** Preoperative and postoperative anteroposterior DSA images demonstrating complete aneurysm occlusion (black arrows)

A 72-year-old man was found to have a 5-mm right MCA aneurysm after experiencing a brief, self-resolving visual disturbance that prompted MRI. He had a history of hypertension but no neurological deficits. The aneurysm had been known for 18 months; although the estimated rupture risk was low, the patient ultimately chose surgical treatment after ongoing discussion. Clipping was favored over endovascular therapy given the aneurysm’s morphology (Fig. 5A, B). The potential for intraoperative rupture or stroke, as well as the benefit of eliminating rupture risk, was reviewed in detail.

Anatomical features influencing the surgical plan included a broad Sylvian fissure extending to the orbital wall and a markedly pneumatized frontal sinus, which posed challenges for standard anterolateral approaches (Fig. 5C). A lateral transorbital route was selected (Fig. 5D). The procedure began with a transpalpebral incision and removal of the lateral orbital rim (Fig. 5E). The greater and lesser sphenoid wings were drilled to expose the frontal and temporal dura, which was then opened to enter the Sylvian fissure (Fig. 5F). Intraoperatively, the aneurysm dome was oriented directly toward the surgeon, while the M1 segment needed for proximal control lay immediately behind it (Fig. 5G). Despite these technical considerations, the aneurysm was clipped successfully with preservation of normal vessels and no residual filling (Fig. 5H-K).

The lateral orbital rim was anatomically replaced and secured using low-profile screws; no additional grafts or reconstruction materials were required. The patient was discharged home on postoperative day two with an intact neurological examination.

In this carefully selected case with favorable anatomy, the microscopic TOA proved feasible and effective as an alternative to the mini-pterional route for MCA aneurysm clipping. The patient provided informed consent for the procedure and publication of this case. Institutional Review Board approval was not required for this report.

## Discussion

TOAs to the skull base have evolved significantly since the early twentieth century, beginning with Frazier’s description of the orbital roof craniotomy in 1913, later refined through eyebrow incisions to minimize morbidity and improve cosmetic outcomes [11, 12]. Since then, these minimally invasive techniques have gained increasing attention for accessing skull base structures, including the lateral cavernous sinus wall, Meckel’s cave, petrous carotid artery, and optic nerve, while avoiding the extent of dissection required in traditional transcranial routes [26, 35, 5, 15–17, 22, 23, 33]. Although microsurgical TOAs to neurovascular lesions remain rare and technically demanding, their potential advantages, including reduced temporalis muscle manipulation, limited frontal and temporal lobe retraction, and the possibility of faster postoperative recovery, make them an appealing alternative for carefully selected anterior circulation aneurysms [27, 45].

In this study, we quantitatively assessed the anatomical feasibility and technical constraints of a microscopic TOA for MCA bifurcation aneurysms through direct comparison with the established MP approach. While the MP approach remains the gold standard for MCA aneurysm clipping due to its wide exposure and established track record, the TOA approach demonstrated a different and unique exposure to the optico-carotid cistern and proximal Sylvian fissure [36, 37, 46]. These findings support the notion that, although not universally applicable, the transorbital route may offer a viable alternative in carefully selected cases [36].

Our findings suggest comparable working distance to the MCA bifurcation in both approaches. The MP approach allows significantly greater surgical maneuverability; however, the horizontal angle of attack was similar in comparison, likely because the TOA takes full advantage of the lateral orbitotomy. This provides a more antero-posteriorly oriented trajectory and a more perpendicular geometric angle of approach to the M1 trunk. However, improved geometric alignment alone does not necessarily translate into easier, faster, or safer proximal control, particularly in the setting of complex aneurysm morphology or intraoperative rupture.

This antero-posteriorly oriented trajectory of TOA may be anatomically favorable for aneurysms with laterally projecting domes, allowing dissection along the direction of the M1 trunk in such cases. Its direct line of sight enables precise clip application with minimal brain retraction, favoring small-to-moderate, well-circumscribed lesions without complex branching [32, 36]. In theory, anteriorly directing aneurysms can be managed via the TOA with wide horizontal angle to dissect the dome at both anterior and posterior aspects, however the lenticulostriate perforators can be challenging with the dome obstructing the view. Posteriorly directing aneurysms, also, might be not suitable for a TOA, given the unfavorable access angle to the aneurysm neck.

Beyond anatomical access, the TOA may offer practical benefits by avoiding the drawbacks commonly associated with transcranial routes, such as temporalis muscle dissection, postoperative muscle atrophy, and visible scarring [21, 34, 42, 46]. Its relatively less disruptive profile may further reduce postoperative pain, edema, and functional impairment, reinforcing its potential role in specific clinical scenarios where both anatomical access and cosmetic outcomes are critical [30, 37, 41, 45]. Nevertheless, the notion of minimal invasiveness in neurosurgery is inherently complex and multifactorial. Although the microscopic TOA obviates temporalis dissection and utilizes a concealed eyelid incision, overall invasiveness must also account for operative exposure, surgical maneuverability, brain retraction, neurovascular manipulation, and the balance of functional and cosmetic outcomes. Other minimally invasive keyhole approaches, such as supraorbital or lateral supraorbital routes, exist and offer related benefits, though they are less commonly applied for MCA aneurysms and differ in trajectory and surgical freedom.

In clinical practice, meticulous preoperative imaging review, particularly utilizing trajectory-based reconstructions and virtual simulations, can significantly enhance surgical planning and approach selection. Virtual reality (VR) based platforms can provide dynamic, trajectory-guided visualization that allows surgeons to assess whether adequate exposure and safe clip deployment can be achieved via the transorbital corridor [27, 41]. Notably, Piper et al. conducted a VR simulation study analyzing 25 unruptured MCA bifurcation aneurysms and found that only 42% were accessible through the TOA. Their findings suggest that aneurysm morphology, including neck width (> 5.5 mm), height (> 7 mm), and overall size (> 8 mm), plays a critical role in approach feasibility. Among these variables, aneurysm width was the only factor that reached statistical significance, as increased width was associated with reduced visibility of the aneurysm neck within the narrow TOA working corridor [10, 36].

Despite its keyhole design, the TOA remains technically demanding and carries several important limitations [24, 13, 25, 28, 43, 44, 47]. Its inherently narrow operative corridor restricts instrument maneuverability and reduces the working angle for clip placement, making complex or broad-neck aneurysms less suitable [31]. The keyhole trajectory can limit visualization of adjacent perforators and deep neurovascular structures, increasing the risk of incomplete exposure. Duraplasty through the TOA may be challenging, raising the potential for postoperative CSF leak [38]. In the event of intraoperative rupture, rapid proximal control of the M1 segment or ICA is essential, and temporary clip placement may be more technically demanding due to the restricted corridor. Surgeons must maintain meticulous microsurgical technique, use small and angled instruments, and be prepared to convert to a standard mini-pterional or orbitozygomatic approach if bleeding cannot be controlled. The TOA also presents inherent technical challenges and a steep learning curve. Consequently, the surgeon’s prior experience and familiarity with the approach are critical determinants of both procedural safety and overall effectiveness [13, 30, 32]. Mastery of delicate maneuvers, precise orientation within a limited corridor, and anticipation of anatomical variations all contribute to achieving optimal outcomes and minimizing complications.

Our results suggest that the TOA may serve as an alternative approach for carefully selected, small-to-moderate MCA aneurysms with lateral, anterosuperior, or anteroinferior projections and relatively simple branching patterns, though it is not broadly generalizable. The narrow operative corridor limits intraoperative instrument adjustment and provides little margin for error, necessitating meticulous surgical planning and advanced microsurgical skill. Multidirectional maneuverability remains superior with the MP approach, reaffirming its role as the gold standard for MCA aneurysm clipping. Future directions should focus on developing robust, anatomy-based selection algorithms that consider aneurysm width, dome projection, neck orientation, and Sylvian fissure accessibility. Patient-specific 3D modeling and virtual surgical simulations may further optimize preoperative planning and help identify cases most suitable for this keyhole corridor.

### Limitations

This study was performed using human cadaveric specimens, which, although anatomically accurate, do not replicate the physiological conditions encountered during live surgery. Key intraoperative variables, such as brain turgor, vascular compliance, pulsation, aneurysm rupture risk, and bleeding, cannot be adequately simulated in cadaveric models and may significantly influence surgical maneuverability and decision-making. Additionally, the absence of true aneurysmal pathology limits the assessment of clip application dynamics, parent vessel manipulation, and the real-time judgment required in complex aneurysm clipping. Potential complications specific to the transorbital approach, such as dural closure, cerebrospinal fluid drainage management and orbital traction injuries, could not be evaluated in this model. Accordingly, the quantitative measurements presented in this study should be interpreted as descriptive anatomical tendencies that inform geometric feasibility and comparative exposure, rather than as predictors of clinical safety, efficiency, or operative outcomes.

## Conclusion

The TOA provides a corridor offering different and unique access to the proximal Sylvian fissure, enabling treatment of select MCA aneurysms. It provides more perpendicular access to the M1 which can be advantageous in proximal control. Carefully selected MCA aneurysms which are small in size with lateral, anterosuperior, or anteroinferior dome orientation may be effectively managed via the TOA. However, the MP approach remains as the gold standard in MCA bifurcation aneurysms. Future work may focus on patient-specific, anatomy-based selection algorithms incorporating 3D modeling and virtual simulations to optimize preoperative planning.

## Data Availability

No datasets were generated or analysed during the current study.

## Abbreviations

TOA: Transorbital approach
ICA: Internal carotid artery
MCA: Middle cerebral artery
MP: Mini-pterional approach
VR: Virtual reality
SOF: Superior orbital fissure

## References

[1] Agosti E et al (2021) Quantitative anatomic comparison of microsurgical transcranial, endoscopic endonasal, and transorbital approaches to the spheno-orbital region. Oper Neurosurg 21(6):E494–E505. 10.1093/ons/opab310 34467999 10.1093/ons/opab310

[2] Agosti E et al (2022) Quantitative anatomic comparison of endoscopic transnasal and microsurgical transcranial approaches to the anterior cranial fossa. Oper Neurosurg 23(4):E256–E266. 10.1227/ons.0000000000000312 36106936 10.1227/ons.0000000000000312

[3] Almeida JP, Ruiz-Treviño AS, Shetty SR, Omay SB, Anand VK, Schwartz TH (2017) Transorbital endoscopic approach for exposure of the sylvian fissure, middle cerebral artery and crural cistern: an anatomical study. Acta Neurochir (Wien) 159(10):1893–1907. 10.1007/s00701-017-3296-8 28808799 10.1007/s00701-017-3296-8

[4] Alves-Belo JT et al (2019) Lateral transorbital versus endonasal transpterygoid approach to the lateral recess of the sphenoid sinus-a comparative anatomic study. Oper Neurosurg 16(5):600–606. 10.1093/ons/opy211 30107582 10.1093/ons/opy211

[5] Beseoglu K, Lodes S, Stummer W, Steiger HJ, Hänggi D (2011) The transorbital keyhole approach: early and long-term outcome analysis of approach-related morbidity and cosmetic results. J Neurosurg 114(3):852–856. 10.3171/2010.9.JNS1095 21029037 10.3171/2010.9.JNS1095

[6] Cheng CM, Dogan A (2019) Quantitative measurement of the surgical freedom for anterior communicating artery complex—a comparative study between the frontotemporal pterional and supraorbital craniotomy; a laboratory study. Acta Neurochir (Wien) 161(12):2513–2519. 10.1007/s00701-019-04097-8 31650332 10.1007/s00701-019-04097-8

[7] Corecha Santos R et al (2024) Exploring optimal microscopic keyhole access to the skull base: an anatomical evaluation of transciliary supraorbital and transpalpebral orbitofrontal craniotomy approaches. Neurosurg Rev 47(1):10.1007/s10143-024-02554-2 10.1007/s10143-024-02554-2 PMC11249509 39009883

[8] Corrivetti F, de Notaris M, Seneca V, Di Nuzzo G, Catapano G (2024) Is it time for a paradigm shift in the surgical management of trigeminal schwannomas? Evaluating the role of the transorbital approach: an anatomo-clinical study and systematic literature review. World Neurosurg. 10.1016/j.wneu.2024.08.055 39151698 10.1016/j.wneu.2024.08.055

[9] Corvino S et al (2024) Assessing the feasibility of selective piezoelectric osteotomy in transorbital approach to the middle cranial fossa: anatomical and quantitative study and surgical implications. World Neurosurg. 10.1016/j.wneu.2024.09.066 39303974 10.1016/j.wneu.2024.09.066

[10] Di Somma A et al (2022) Endoscopic transorbital surgery levels of difficulty. J Neurosurg 137(4):1187–1190 35426817 10.3171/2022.3.JNS212699

[11] Frazier CH (1913) An approach to the hypophysis through the anterior cranial fossa. Ann Surg 57(2):145–150 17862963 10.1097/00000658-191302000-00001 PMC1407403

[12] Gulsuna B et al (2024) Endoscopic endonasal approach to the orbit: a case series and clinical experience emphasizing the advantages of the ipsilateral mononostril technique. World Neurosurg 186:e273–e282 38548053 10.1016/j.wneu.2024.03.122

[13] Harris P et al (2025) Comparison of patient-centric factors in minimally invasive transcranial versus classic approaches: a matched cohort study. J Neurosurg 1(aop):1–11 10.3171/2025.2.JNS242843 40479818

[14] Houlihan LM et al (2024) Quantitative analysis of the supraorbital, transorbital microscopic, and transorbital neuroendoscopic approaches to the anterior skull base and paramedian vasculature. J Neurol Surg B Skull Base 86(3):313–324. 10.1055/s-0044-1786373 40351872 10.1055/s-0044-1786373 PMC12064300

[15] Houlihan LM, Belykh E, Zhao X, O’Sullivan MGJ, Preul MC (2021) From Krönlein, through madness, to a useful modern surgery: the journey of the transorbital corridor to enter the neurosurgical armamentarium. J Neurosurg 135(4):1270–1279. 10.3171/2020.8.JNS201251 33545682 10.3171/2020.8.JNS201251

[16] Houlihan LM et al (2023) Transorbital microsurgery: an anatomical description of a minimally invasive corridor to the anterior cranial fossa and paramedian structures. J Neurol Surg B Skull Base 85(5):470–480. 10.1055/s-0043-1772202 39233771 10.1055/s-0043-1772202 PMC11368469

[17] Houlihan LM et al (2021) Transorbital neuroendoscopic surgery as a mainstream neurosurgical corridor: a systematic review. World Neurosurg. 10.1016/j.wneu.2021.04.104 33940270 10.1016/j.wneu.2021.04.104

[18] Jean WC, Patrick HH, Najera E (2024) Minimally invasive lateral transorbital approach for clipping of right middle cerebral artery aneurysm: 2-dimensional operative video. Oper Neurosurg. 10.1227/ons.0000000000001291 39037229 10.1227/ons.0000000000001291

[19] Jean WC, Piper K, Wojcik R, Saez-Alegre M (2023) Transorbital approach for clipping of anterior communicating artery aneurysm: 2-dimensional operative video. Oper Neurosurg 25(4):E237. 10.1227/ons.0000000000000816 37499257 10.1227/ons.0000000000000816

[20] Jean WC, Rubino F, Harris P, Sàez-Alegre M (2025) Minimally invasive transorbital approach for anterior communicating artery aneurysms: how i do it. Acta Neurochir (Wien). 10.1007/s00701-025-06556-x 40343575 10.1007/s00701-025-06556-x PMC12064573

[21] Jean WC, Sack KD, Tsen AR (2022) Augmented-reality template guided transorbital approach for intradural tumors. Neurosurgical Focus: Video. 10.3171/2021.10.FOCVID21172 36284590 10.3171/2021.10.FOCVID21172 PMC9555352

[22] Jeon C et al (2025) Endoscopic transorbital approach for resection of mediobasal temporal lesions using an optic radiation–sparing strategy. J Neurosurg 142(3):819–828. 10.3171/2024.6.JNS232810 39454213 10.3171/2024.6.JNS232810

[23] Jung IH et al (2022) Endoscopic transorbital approach to the cavernous sinus: cadaveric anatomy study and clinical application (SevEN-009). Front Oncol. 10.3389/fonc.2022.962598 36091168 10.3389/fonc.2022.962598 PMC9459324

[24] Karımzada G et al (2024) Transorbital neuroendoscopic surgery for treatment of sphenoid wing meningiomas extending to the cavernous sinus: clinical implications and a technical illustration. Neurosurg Focus 56(4):E810.3171/2024.1.FOCUS23857 38560930 10.3171/2024.1.FOCUS23857

[25] Kim W, Moon JH, Kim EH, Hong CK, Han J, Hong JB (2021) Optimization of orbital retraction during endoscopic transorbital approach via quantitative measurement of the intraocular pressure – [SevEN 006]. BMC Ophthalmol. 10.1186/s12886-021-01834-5 33557770 10.1186/s12886-021-01834-5 PMC7871604

[26] Komaitis S et al (2024) The lateral retrocanthal transorbital endoscopic approach to the middle fossa: cadaveric stepwise approach and review of quantitative cadaveric data. Neurosurg Focus. 10.3171/2024.1.FOCUS23839 38560924 10.3171/2024.1.FOCUS23839

[27] Kong DS, Lee WJ, Kim GJ, Hong CK (2025) The feasibility and clinical outcome of endoscopic transorbital transcavernous approaches with or without petrosectomy for petroclival lesions. J Neurosurg 142(4):1058–1065. 10.3171/2024.6.JNS232976 39504547 10.3171/2024.6.JNS232976

[28] Kong DS et al (2019) Clinical and ophthalmological outcome of endoscopic transorbital surgery for cranioorbital tumors. J Neurosurg 131(3):667–675. 10.3171/2018.3.JNS173233 30215555 10.3171/2018.3.JNS173233

[29] Kurbanov A, Sanders-Taylor C, Keller JT, Andaluz N, Zuccarello M (2015) The extended transorbital craniotomy: an anatomic study. Oper Neurosurg 11(2):338–344. 10.1227/NEU.0000000000000762 10.1227/NEU.0000000000000762 25867616

[30] López CB et al (2021) Extradural anterior clinoidectomy through endoscopic transorbital approach: laboratory investigation for surgical perspective. Acta Neurochir (Wien) 163(8):2177–2188. 10.1007/s00701-021-04896-y 34110491 10.1007/s00701-021-04896-y

[31] Mandel M, Tutihashi R, Mandel SA, Teixeira MJ, Figueiredo EG (2017) Minimally invasive transpalpebral ‘Eyelid’ approach to unruptured middle cerebral artery aneurysms. Oper Neurosurg 13(4):453–464. 10.1093/ons/opx021 28838124 10.1093/ons/opx021

[32] Mao G, Gigliotti M, Aziz K, Aziz K (2020) Transpalpebral approach ‘eyelid incision’ for surgical treatment of intracerebral aneurysms: lessons learned during a 10-year experience. Oper Neurosurg 18(3):309–315. 10.1093/ons/opz217 31414139 10.1093/ons/opz217

[33] Moe KS, Bergeron CM, Ellenbogen RG (2010) Transorbital neuroendoscopic surgery. Oper Neurosurg 67(3):ons16–ons28 10.1227/01.NEU.0000373431.08464.43 20679952

[34] Mosteiro A et al (2025) The transorbital approach to the internal carotid and middle cerebral arteries. A dissection study toward targeted access aneurysm clipping. World Neurosurg 194:123486 39577657 10.1016/j.wneu.2024.11.069

[35] Park HH, Yoo J, Yun IS, Hong CK (2020) Comparative analysis of endoscopic transorbital approach and extended mini-pterional approach for sphenoid wing meningiomas with osseous involvement: preliminary surgical results. World Neurosurg 139:e1–e12. 10.1016/j.wneu.2020.01.115 32001400 10.1016/j.wneu.2020.01.115

[36] Piper K et al (2024) Transorbital approach clipping of middle cerebral artery aneurysm: a virtual reality morphometric anatomic study. World Neurosurg. 10.1016/j.wneu.2024.08.152 39236805 10.1016/j.wneu.2024.08.152

[37] Plata-Bello J et al (2024) The endoscopic transorbital approach for vascular surgery: an anterior circulation anatomic study, 2-dimensional operative video. Oper Neurosurg. 10.1227/ons.0000000000001254 38967432 10.1227/ons.0000000000001254

[38] Ricciuti V et al (2024) Endoscopic transorbital approach for recurrent spheno-orbital meningiomas: single center case series. Neurosurg Rev 47(1):706. 10.1007/s10143-024-02905-z 39348070 10.1007/s10143-024-02905-z PMC11442621

[39] Saraceno G et al (2020) Quantitative anatomical comparison of anterior, anterolateral and lateral, microsurgical and endoscopic approaches to the middle cranial fossa. World Neurosurg 134:e682–e730. 10.1016/j.wneu.2019.10.178 31731015 10.1016/j.wneu.2019.10.178

[40] Serioli S et al (2023) Surgical anatomy of the microscopic and endoscopic transorbital approach to the middle fossa and cavernous sinus: anatomo-radiological study with clinical applications. Cancers (Basel). 10.3390/cancers15184435 37760405 10.3390/cancers15184435 PMC10527149

[41] Steiger H-J, Schmid-Elsaesser R, Stummer W, Uhl E (n.d.) Transorbital keyhole approach to anterior communicating artery aneurysms.” [Online]. Available: http://journals.lww.com/neurosurgery. Accessed 08/02/2025 10.1097/00006123-200102000-00021 11220378

[42] Ulutas M, Çinar K, Dogan I, Secer M, Isik S, Aksoy K (2021) Lateral transorbital approach: an alternative microsurgical route for supratentorial cerebral aneurysms. J Neurosurg 134(1):72–83. 10.3171/2019.9.JNS191683 31783357 10.3171/2019.9.JNS191683

[43] Vural A et al (2021) Transorbital endoscopic approaches to the skull base: a systematic literature review and anatomical description. Neurosurg Rev. 10.1007/s10143-020-01470-5 33479806 10.1007/s10143-020-01470-5 PMC8490260

[44] Vural A et al (2021) Transorbital endoscopic approaches to the skull base: a systematic literature review and anatomical description. Neurosurg Rev. 10.1007/s10143-020-01470-5 33479806 10.1007/s10143-020-01470-5 PMC8490260

[45] Wang H et al (2015) Clipping of anterior communicating artery aneurysms in the early post-rupture stage via transorbital keyhole approach - Chinese neurosurgical experience. Br J Neurosurg 29(5):644–649. 10.3109/02688697.2015.1023774 25968329 10.3109/02688697.2015.1023774

[46] Yoo J, Park HH, Yun I-S, Hong C-K (2021) Clinical applications of the endoscopic transorbital approach for various lesions. Acta Neurochir. 10.1007/s00701-020-04694-y 33394139 10.1007/s00701-020-04694-y

[47] Zhao X et al (2024) Novel eyelid supraorbital pretemporal approach to the anterior communicating artery complex: a quantitative cadaveric comparative study. J Neurol Surg B Skull Base. 10.1055/a-2324-9499 40620636 10.1055/a-2324-9499 PMC12227245

[48] Zhao X et al (2025) The eyelid trans-orbital trans-cavernous approach to the basilar apex: a cadaveric proof-of-concept study. J Neurol Surg B Skull Base 86(03):325–331 40351875 10.1055/s-0044-1787148 PMC12064301

